# The effect of familiarization with preoperative care on anxiety and vital signs in the patient’s cesarean section: A randomized controlled trial

**DOI:** 10.18332/ejm/137366

**Published:** 2021-06-25

**Authors:** Mehrnush Mostafayi, Behzad Imani, Shirdel Zandi, Faeze Jongi

**Affiliations:** 1 Student Research Center, Hamadan University of Medical Sciences, Hamadan, Iran; 2 Department of Operating Room, Faculty of Paramedicine, Hamadan University of Medical Sciences, Hamadan, Iran

**Keywords:** anxiety, cesarean section, familiarization, vital signs

## Abstract

**INTRODUCTION:**

Cesarean section (C-section) is one of the most prevalent surgeries among women. The preoperative stages in the surgery day and lack of control over being in an unfamiliar situation and feeling danger cause anxiety, and consequently, instability in patients. This study aimed to determine the effect of familiarization with preoperative nursing care on anxiety and vital signs of patients in cesarean section.

**METHODS:**

This randomized controlled trial study was performed on 80 pregnant candidates for C-section in Hamadan Fatemieh Hospital, Iran, in 2020. Patients were randomly divided into control (n=40) and intervention (n=40) groups. The control group just received the routine intervention of the hospital, but the intervention group, in addition, received the two familiarizing sessions with preoperative nursing care. Data were collected via vital signs sheet and Spielberger situational anxiety questionnaire and were analyzed using SPSS16 software at a significance level of p=0.05.

**RESULTS:**

Before the intervention, there was no significant difference between the mean anxiety scores of the control and intervention groups, and the two groups were homogeneous (p=0.396). However, after the intervention, the mean anxiety of the intervention group decreased significantly (p=0.001) and increased in the control group (p=0.600); and the mean post-test of the two groups showed a significant difference (p=0.001). After the intervention, the mean heart rate, respiration rate, systolic and diastolic blood pressure in the intervention group decreased significantly (p<0.05). However, there was no significant difference in heart rate and systolic blood pressure of the control group (p>0.05).

**CONCLUSIONS:**

Based on the results of this study we conclude that familiarity with preoperative care reduces the level of anxiety and stabilized the level of vital signs parameters.

## INTRODUCTION

Cesarean section (C-section) is one of the most prevalent surgeries in women^1^. Today, despite the emphasis on vaginal delivery and the use of extensive media promotion and special facilities devoted for this purpose, the number of C-sections is increasing around the world and is higher than the recommended rate of the World Health Organization (10–15%)^2^. According to reports from different countries, the frequency of C-section is more than 18% worldwide^3^. A meta-analysis study in Iran estimated the prevalence of C-section at 48%^4^. Maternal complications include bleeding, pain, post-operative ileus, incision infection^5,6^, fetal complications, transient tachypnea of the newborn, respiratory distress syndrome, hospitalization in the neonatal intensive care unit, and wounds caused by surgical blades^7^. Other complications are anxiety and changes in vital signs during labor^8^.

Anxiety is a state of fear in patients that arises from the prediction of a threatening event. Anxiety is common in surgical candidates. Cesarean candidates experience higher levels of anxiety than patients ready for surgery^9^. C-section can cause irreversible risks to the mother, baby and fetus^10^ and is a stressful surgery^11^. Feelings of helplessness and low self-esteem, stress, and anxiety have been reported frequently among C-section women^10^. Previous studies have shown that 30.9% of mothers will experience anxiety during pregnancy^12^. The main reasons for preoperative anxiety are doubts about the success of surgery and fear of the threats that surgery can entail^13^. The effects of prenatal anxiety include enhanced levels of cortisol, anti-inflammatory cytokines, and less breastfeeding. Its effects on infants also include preterm delivery^14^. Anxiety also causes symptoms such as increased blood pressure, heart rate, and respiration in mother by stimulation of the autonomic nervous system^8^.

Patient anxiety can be controlled with pharmacological and non-pharmacological interventions^15^. Anxiety medications are commonly used to control the side effects. Routine administration of these drugs delays the mother’s recovery after the C-section, due to its sedative and nauseating effects. Therefore, they inhibit the early mother–infant relationship. They also reduce the mother’s ability to start effective feeding^16^. Today, there are various non-pharmacological methods to reduce anxiety and to control changes in mothers’ vital signs during childbirth. For example, music therapy^17^ and reflexology are some of the methods used in prenatal and postnatal nursing care^18^.

Nowadays, familiarization and education are among the most basic care programs in the health care system, i.e. responding to the patients’ learning needs and patients’ education^19^. Education about preoperative preparation, anaesthetizing process, control of complications such as nausea, vomiting, and postoperative pain, and how to care for the patient during the operation can fill the information gap among the patients and consequently reduce their anxiety^20^. Therefore, this study aimed to determine the effect of familiarization with preoperative nursing care on anxiety and vital signs of mothers undergoing C-section.

## METHODS

### Study design

In this study, a controlled clinical trial method was used to determine the effect of familiarization with preoperative nursing care strategies on anxiety and vital signs among the patients undergoing C-section.

### Participants and setting

**Figure 1:**
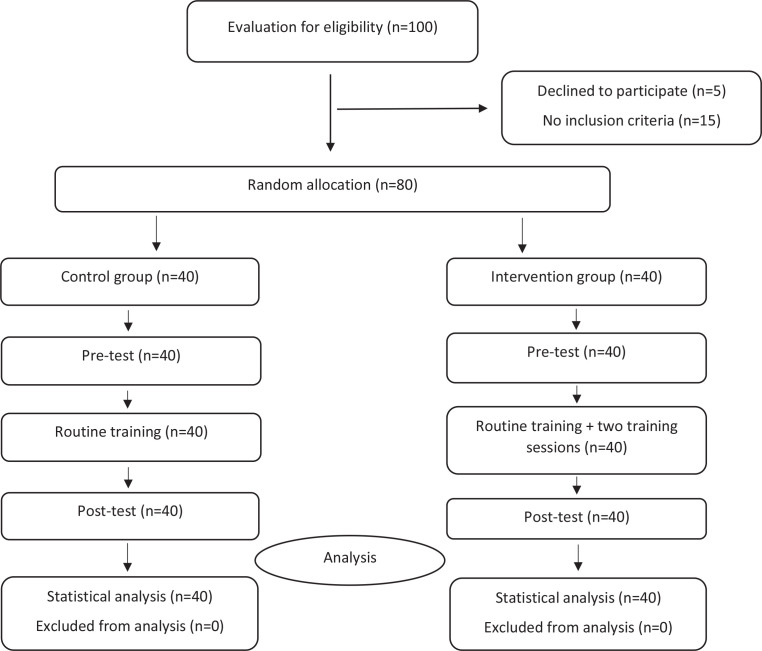
CONSORT flow diagram of participants

The samples were selected via convenience sampling method among those patients that referred to the Hamadan Fatemieh Hospital, Iran, for the C-section, from June to July 2020. Inclusion criteria were: candidate for non-emergency C-section, gestational age over 34 weeks, with clear consciousness, lack of psychological problems, singleton term and full-term pregnancy, and 12 to 24 hours from hospital admission to operating room admission. Exclusion criteria were: aged <16 years and >45 years, complicated anesthesia, preterm delivery, need for emergency C-section, women critical conditions including fetal distress, placental abruption, uterine rupture, severe eclampsia, severe pregnancy-induced hypertension, extensive bleeding intraoperative (>2000 mL), and underlying diseases such as renal, cardiovascular disorders. After the patients signed the informed consent form, the quadratic block method (Block Randomization) was used to devote them randomly in the two equal groups. The letters I and C meaning ‘Intervention’ and ‘Control’, respectively, were written on two completely identical lottery cards, and the cards were placed next to each other in the desk drawer. One member of the research team was responsible for sequencing randomization at the beginning of the study. Sample size was determined according to Talaie et al.^21^. Considering d=1, 1-α=99%, 1-β=90%, and significance level=0.05, the sample size was estimated at 80 for the study (Figure 1).

### Intervention

Mothers were initially informed about the objectives of the study and ensured of issues of confidentiality. They then had to sign a written informed consent form to participate in the research and complete the demographic information form. Before any intervention during hospital admission as a pretest, vital signs of all patients were recorded and the level of situational anxiety was measured. The control group just received the usual hospital intervention. In the intervention group, in addition to the routine intervention of the hospital, two sessions of introductory program were held for familiarization with preoperative nursing care (Table 1); each session lasted one hour, and the patient was trained face-to-face by the researcher. In this study, patients were familiarized with preoperative nursing care based on an educational package, this package was prepared based on the opinion of nursing experts and its content validity was examined. After the intervention and when the patient was admitted to the operating room, the vital signs and anxiety levels of all patients were evaluated as a post-test.

**Table 1:** Timing and objectives of the intervention sessions

*Session*	*Objectives*
**First**	Preoperative preparation, prevention of preoperative hypothermia, post-operative pain relief, prevention and treatment of post-operative nausea and vomiting
**Second**	Prevention of surgical site infection, postpartum hemorrhage, sexual dysfunction, baby care and breastfeeding

### Demographic and Spielberger situational anxiety questionnaire

Demographic information included age, gestational age, level of education, and occupation. The Spielberger anxiety questionnaire was used to assess anxiety levels. It specifies the person’s immediate anxiety, i.e. a person’s current feelings in a time span, such as preparing time for surgery. During previous studies, the validity and reliability of this scale was determined. According to Mahram^22^, to achieve the meaningfulness of the result, the average anxiety levels for the standard and normal populations were compared at about 1% and 5% levels for all age groups, which showed the validity of the anxiety measure. Besides, the scientific validity was confirmed using Cronbach’s alpha, which was equal to 0.945^22^. The questionnaire consisted of 20 multiple-choice questions with options: ‘very little’, ‘little’, ‘much’, and ‘very much’. The minimum score of this questionnaire was 20 and the maximum was 80. In this study, the reliability was 0.76 based on the Cronbach’s α formula.

### Digital blood pressure monitor and chronometer

All research units used digital measuring devices to measure blood pressure and heart rate (ALPK digital blood pressure monitor, Japan). A single device was used for all research units. The number of breaths per minute was measured by observing and touching the chest and using a chronometer.

### Statistical analysis

The data collected from two parts of descriptive and inferential statistics were analyzed. In the descriptive part, the distribution of graphs and central indicators and dispersion were applied; and in the inferential part, an independent t-test and paired t-test were used. SPSS (version16) software was used for statistical analysis.

## RESULTS

As shown in Table 2, the two groups were homogeneous in terms of age and gestational age (p>0.05) and in terms of literacy and employment (p>0.05). In the two groups, the literacy level of the patients was mostly below diploma and they were mostly unemployed.

**Table 2:** Table of demographic variables of control and intervention groups

*Variables*		*Control group mean ± SD*	*Intervention group mean ± SD*	*p*
**Age** (years)		30/5 ± 5/31	30.22 ± 5.88	0.827
**Gestational age** (weeks)		37/4 ± 1/87	37.71 ± 1.12	0.180
		***n (%)***	***n (%)***	***p***
**Education level**	Below diploma	32 (80)	26 (65)	0.322
	Diploma	5 (12.5)	9 (22.5)	
	University	3 (7.5)	5 (12.5)	
**Occupation**	Housewife	39 (97.5)	39 (97.5)	0.368
	Freelance	0 (0)	1 (2.5)	
	Employee	1 (2.5)	0 (0)	

As shown in Table 3, the results show that before the intervention, there was no significant difference between the mean anxiety scores of the control and intervention groups, and the two groups were homogeneous (p=0.396). However, after the intervention, the mean anxiety of the intervention group decreased significantly (p=0.001) and increased in the control group, although it was not statistically significant (p=0.600); so that the mean post-test of the two groups showed a significant difference (p=0.001).

**Table 3:** Comparison of intergroup and intragroup anxiety levels of control and intervention groups before and after the intervention

*Variable*		*Control group (n=40) mean ± SD*	*Intervention group (n=40) mean ± SD*	*Independent t-test*
**Anxiety**	Pre-test	48.55 ± 6.04	49.50 ± 3.62	p=0.396, t=-0.85, df=78
	Post-test	47.84 ± 6.51	44.47 ± 3.66	p=0.006, t=2.85, df=78
	Paired t-test	p=0.600, t=0.529, df=39	p=0.001, t=7.594, df=39	

Moreover, as noted in Table 4 before the intervention, there was no significant difference between the control and intervention groups’ mean heart rate, respiration rate, systolic blood pressure, and diastolic blood pressure (p>0.05). After the intervention, the mean heart rate, respiration rate, systolic and diastolic blood pressure in the intervention group decreased significantly (p<0.05). However, there was no significant difference in heart rate and systolic blood pressure of the control group (p>0.05), while the number of breaths and diastolic blood pressure increased significantly (p<0.05). It was also revealed that the two groups after the intervention were significantly different in terms of heart rate, respiration, systolic and diastolic blood pressure, and the values of these variables were less in the intervention group (p<0.05) (Table 4).

**Table 4:** Comparison of intergroup and intragroup rate of vital signs in the control and intervention groups before and after the intervention

*Variables*		*Control group (n=40) mean ± SD*	*Intervention group (n=40) mean ± SD*	*Independent t-test*
**Heart rate**	Pre-test	92.52 ± 11.85	93.10 ± 11.24	p=0.824, t=-0.223, df=78
	Post-test	95.25 ± 13.22	87.05 ± 12.10	p=0.005, t=2.89, df=78
	Paired t-test	p=0.265, t= -1.13, df=39	p=0.002, t=3.31, df=39	
**Respiration rate**	Pre-test	20.02 ± 8.38	19.52 ± 2.45	p=0.718, t=0.362, df=78
	Post-test	22.77 ± 4.39	17.85 ± 3.66	p=0.001, t=5.44, df=78
	Paired t-test	p=0.046, t=39, df=-2.05	p=0.027, t=39, df=2.29	
**Systolic blood pressure**	Pre-test	124.68 ± 17.55	125.98 ± 14.27	p=0.717, t=-0.363, df=78
	Post-test	126.55 ± 14.60	120.42 ± 7.61	p=0.021, t=2.35, df=78
	Paired t-test	p=0.512, t=-0.66, df=39	p=0.035, t=2.17, df=39	
**Diastolic blood pressure**	Pre-test	74.75 ± 9.61	75.65 ± 9.81	p=0.680, t=-0.414, df=78
	Post-test	79.07 ± 14.28	69.62 ± 11.14	p=0.001, t=3.29, df=78
	Paired t-test	p=0.025, t=-2.33, df=39	p=0.005, t=-2.33, df=39	

## DISCUSSION

The aim of this study was to determine the effect of familiarization with preoperative nursing care on anxiety and vital signs of patients undergoing C-section on 80 patients referred to Hamadan Fateimeh Hospital, Iran. The results of this study clearly proved the positive effect of familiarizing the patients with preoperative nursing care.

This study showed that patients who are familiar with preoperative care will experience less anxiety when admitted to the operating room. This is very important for patients because this method can be used as a non-pharmacological and non-invasive strategy, for mothers to experience less anxiety from the operating room and C-section. Similar studies, such as the Akbarzadeh et al.^23^ have proved that the nurse companionship with the patient has a positive effect on reducing maternal anxiety during and after C-section, which is consistent with the results of the present study. Also, the Shirdel et al.^24^ study, conducted as a randomized clinical trial on 90 patients volunteering for CABG surgery, emphasized that preoperative counseling and admission to the operating room by a counselor can reduce anxiety levels and stabilize patients’ vital signs. Also, a study by Dehghani et al.^25^ conducted in 2013 in Yazd on 100 patients volunteering for CABG surgery, showed that the mean anxiety score of the experimental group significantly decreased after the intervention. Moreover, in the study of Roshangar et al.^26^, which was conducted as a randomized clinical trial study, they concluded that the nurse’s companionship has a positive effect on reducing maternal anxiety during and after C-section. Therefore, this method can be used as a non-invasive care during the C-section and in operating rooms. Also, in the study of Eslami et al.^27^ as a randomized controlled clinical trial study, they emphasized that the familiarity of patients with C-section and operating room environment reduced the level of anxiety before C-section and reduced post-operative pain. Further, this non-pharmacological method of controlling anxiety before surgery can have a positive effect on the post-operative recovery of patients, because it has been shown that high levels of anxiety before surgery can negatively affect patients’ recovery from anesthesia and post-operative pain control^13^.

In this study, we identified that familiarizing the patient with the planned care, in addition to reducing patients’ anxiety, can also reciprocally affect hemodynamic parameters and bring these parameters closer to the normal level. In the present study, it was shown that heart rate, respiration rate, systolic and diastolic blood pressure in patients who were familiar with preoperative care were significantly reduced compared to those among the control group and closer to normal levels. The results of tests comparing the mean pretest score of vital signs in the control and intervention groups show that there is no significant difference between the mean heart rate, respiration rate, and systolic and diastolic blood pressure in the two groups. This finding is consistent with the results of the study of Adib-Hajbaghery et al.^28^ on the lack of significant differences between heart rate variables, respiratory rate, and systolic blood pressure in the control and intervention groups. It is also consistent with the results of the study of Orujlu et al.^29^ which showed that there is no significant difference between the mean vital signs of control and intervention groups before the intervention.

We identified a significant difference between the mean post-test heart rate of the two groups. Therefore, familiarity with preoperative care reduces heart rate, which is consistent with the results of other studies (Table 5) such as that of Orujlu et al.^29^ which showed the effectiveness of interventions on reducing heart rate in the experimental group. However, in contrast to these results, García Sierra et al.^30^ compared two interventions including support based on behavioral education and providing information and positive reinforcement during the procedure, and support based only on providing information on the vital signs of gastroscopic patients found that the mean blood pressure of diastole and systole and pulse were not significantly different between the two groups. According to the results, there is a significant difference between the mean post-test number of breaths between the two groups, which is in line with the results of the study of Hemmati et al.^31^ which showed a significant difference in the mean number of breaths between the control and intervention groups after the intervention. There is a significant difference between the mean post-test of systolic blood pressure between the two groups. Therefore, familiarity with preoperative care reduces systolic blood pressure. It is consistent with the results of studies such as the study of Adib-Hajbaghery et al.^28^, which showed a significant difference between the mean systolic blood pressure of the control and experimental groups after the intervention. However, this finding contradicts the results of the study of García Sierra et al.^30^. According to the results, there is a significant difference between the mean post-test of diastolic blood pressure between the two groups. Therefore, familiarity with preoperative care reduces diastolic blood pressure. However, it is in contrast to the study of Orujlu et al.^29^, which showed the ineffectiveness of interventions on diastolic blood pressure in the intervention group, and the difference between it and the control group is not significant.

**Table 5:** Comparison of the most important findings of the present study with previous studies

*Variables*	*Result of this study*	*Result of previous studies (first author, date)*	*Compare*
**Anxiety**	Familiarization with preoperative nursing care can significantly reduce the anxiety.	Roshangar (2020), the nurse companionship can significantly reduce the anxiety.	Consistent
		Eslam (2020), familiarity of patients with cesarean section and operating room environment can significantly reduce the anxiety.	Consistent
**Heart rate**	Familiarization with preoperative nursing care can significantly reduce the level of heart rate.	Orujlu (2014), nurses’ non-pharmacological interventions can significantly reduce the level of heart rate.	Consistent
		Garcia Sierra (2013), nurses’ non-pharmacological interventions cannot significantly reduce the level of heart rate.	Contrary
**Breath rate**	Familiarization with preoperative nursing care can significantly reduce the level of breath rate.	Hemmati (2013), nurses’ non-pharmacological interventions can significantly reduce the level of breath rate.	Consistent
		Zandi (2020), nurses’ non-pharmacological interventions can significantly reduce the level of breath rate.	Consistent
**Systolic blood pressure**	Familiarization with preoperative nursing care can significantly reduce the level of systolic blood pressure.	Haj Bagheri (2014), nurses’ non-pharmacological interventions can significantly reduce the level of systolic blood pressure.	Consistent
		Garcia Sierra (2013), nurses’ non-pharmacological interventions cannot significantly reduce the level of systolic blood pressure.	Contrary
**Diastolic blood pressure**	Familiarization with preoperative nursing care can significantly reduce the level of diastolic blood pressure.	Orujlu (2014), nurses’ non-pharmacological interventions can significantly reduce the level of diastolic blood pressure.	Consistent
		Zandi (2020), nurses’ non-pharmacological interventions cannot significantly reduce the level of diastolic blood pressure.	Contrary

## CONCLUSIONS

According to the findings of the study, familiarity with preoperative care decreases the level of anxiety and stabilizes the patients’ vital signs, so perinatal healthcare providers can play an effective role in reducing pregnant women’s anxiety. It is suggested that C-section surgery centers adopt programs for identifying strategies to reduce anxiety and stabilize the vital signs of pregnant women.
